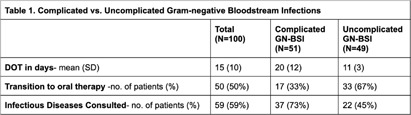# Epidemiology and Duration of Therapy in Patients with Gram-negative Bloodstream Infections: Retrospective Analysis

**DOI:** 10.1017/ash.2024.155

**Published:** 2024-09-16

**Authors:** Hawra Al Lawati, Ellen Cook, Matthew Lee

**Affiliations:** Beth Israel Deaconess Medical Center

## Abstract

**Background:** Longer courses of antibiotics can be associated with antimicrobial resistance and adverse effects. Randomized clinical trials support treating gram-negative bloodstream infections (GN-BSI) for a shorter duration with a consensus that a seven-day course of antibiotics is appropriate for uncomplicated GN-BSI. Prior to the implementation of a GN-BSI treatment guideline at our institution, we aimed to evaluate the characteristics of patients with GN-BSI and the duration of antibiotic therapy (DOT). **Method:** We retrospectively reviewed adult inpatients who had a blood culture with at least 1 gram-negative organism within 6 months (November 2022 to April 2023). Patients were excluded if they had a concomitant gram-positive bloodstream infection or if they were transitioned to comfort-focused care within 48 hours of their first positive blood culture. Complicated GN-BSI was defined as exhibiting any of the following: involvement of bone, joint, endovascular system, or foreign body, an inability to achieve source control, immunocompromised status, or failure to demonstrate clinical improvement or culture clearance within 72 hours. The primary outcome of this study was the mean DOT in patients with GN-BSI. **Result:** 100 patients met the inclusion criteria. Escherichia coli, identified in 54 cases, emerged as the most frequent organism. Urine (41) was the predominant source of bacteremia. Cefepime (48) was the most common empiric agent used. Of the 91 patients with available ceftriaxone susceptibility results, 84% had a susceptible organism. Amongst the 51 patients classified as having a complicated GN-BSI, the leading reason was immunosuppression. Table 1 presents a comparative analysis of complicated vs. uncomplicated GN-BSI. The average DOT for complicated GN-BSI was longer than the uncomplicated infections (20 vs. 11 days, P < 0 .005). Additionally, fewer patients transitioned to oral therapy in the complicated group (33% vs. 67%, P < 0 .005). **Conclusion:** At our institution, patients with uncomplicated GN-BSI have a shorter DOT and are more likely to transition to oral therapy than those with complicated GN-BSI. However, the mean DOT for uncomplicated infections remained longer than seven days and a large number of uncomplicated GN-BSI patients did not transition to oral therapy, indicating room for improvement in local practice through antimicrobial stewardship initiatives.

**Figure:**